# Speckle Measurement for Small In-Plane Vibration Using GaAs

**DOI:** 10.3390/s23052724

**Published:** 2023-03-02

**Authors:** Jiongye Gao, Bin Zhang, Qibo Feng, Xu Shen, Yong Xue, Jiacheng Liu

**Affiliations:** Key Laboratory of Luminescence and Optical Information, Ministry of Education, Beijing Jiaotong University, Beijing 100044, China

**Keywords:** in-plane vibration, GaAs, speckle measurement, photoinduced electromotive force

## Abstract

In this study, the measurement characteristics of speckles based on the photoinduced electromotive force (photo-emf) effect for high-frequency, small-amplitude, and in-plane vibration were theoretically and experimentally studied. The relevant theoretical models were utilized. A GaAs crystal was used as the photo-emf detector for experimental research, as well as to study the influence of the amplitude and frequency of the vibration, the imaging magnification of the measuring system, and the average speckle size of the measuring light on the first harmonic of the induced photocurrent in the experiments. The correctness of the supplemented theoretical model was verified, and a theoretical and experimental basis was provided for the feasibility of using GaAs to measure in-plane vibrations with nanoscale amplitudes.

## 1. Introduction

The measurement of high-frequency micro-vibrations plays a critical role in multiple areas, such as micro-electro-mechanical systems, structural health monitoring, laser ultrasonic technology, materials science, and biomedicine [1,2,3,4,5,6,7,8]. Optical measurement methods with non-contact characteristics have been widely studied and applied [7,8,9,10]. Compared with the measurement of out-of-plane vibration, where the measuring light is parallel to the vibration direction, the measurement of in-plane vibration is relatively complex because the measuring light cannot be incident on the vibration surface in the direction parallel to the in-plane vibration direction. Speckle interferometry is commonly used for in-plane displacement measurements [11,12,13]. However, owing to the high-speed performance limitation of charge-coupled devices in the imaging process, they cannot measure higher frequencies. In addition, there is a low tolerance for the presence of speckles in laser beams from rough vibration surfaces for some traditional laser interferometers, leading to low measurement sensitivity.

Laser interferometry based on photorefractive crystals has become a research focus because of its advantages of wavefront matching and low-frequency cutoff [14,15] and offers some potential research directions and applications with many emerging materials [16,17,18,19]. The corresponding vibration measurement methods can restrain the influence of rough surface speckles and environmental disturbances on the measurements, thereby improving the measurement sensitivity. Among them, laser interferometry based on the photoinduced electromotive force (photo-emf) effect was first reported by Stepanov et al. [20]. In this case, the photo-emf effect is usually produced by projecting a non-steady two-beam interference light pattern on a photorefractive crystal. The interference pattern is created by the interference of a probe beam scattered by the measured sample with a coherent reference beam. The vibration of this pattern subsequently produces an ac electric current in the crystal. Stepanov et al. successively conducted theoretical research on the photo-emf effects of different photorefractive materials, as well as experimental research on interferometry under external electric field modulation for out-of-plane vibration measurement [21,22,23,24,25,26,27,28]. Because of the non-steady characteristics of the photo-emf, as long as the beam with harmonic oscillation shines on the photorefractive material, an alternating photocurrent will be generated. This characteristic makes it possible to measure in-plane vibration with a non-interfering speckle beam. Meanwhile, due to the non-reference beam path, it has the advantage of a simple optical arrangement. Mosquera and Frejlich conducted research on the speckle pattern measurement of in-plane vibration using a BTO crystal, and they proposed that the photocurrent signal can reach a maximum value under a certain vibration amplitude [29]. Santos conducted a theoretical study on the photocurrent signal generated by a transverse-vibration-modulated speckle light incident on a GdTe:V crystal and proposed a corresponding mathematical model and the application of this photo-emf signal to evaluate the response time of the CdTe:V crystal [30,31,32,33]. Heinz and Garmire used an array of semi-insulating GaAs photoconductive sensors to detect intensity variations caused by the transverse movement of speckles [34]. Salazar conducted experimental research based on BSO crystals and analyzed the influence of speckle size on the photocurrent [35]. Bryushinin studied the strains and stresses of their measured medium, caused by mechanical vibration, using a photo-emf sensor and measured the resonant frequency of the mechanical system and the distribution of the phase modulation amplitude on the measured surface [36].

In this study, a speckle measurement method for in-plane vibration based on a GaAs crystal photo-emf was investigated. A concrete calculation of the response time was added to the corresponding theoretical model. With the GaAs crystal as the photo detector, the effects of the vibration amplitude and frequency, as well as those of the imaging magnification of the measurement system and the average speckle size of the measuring light on the induced photocurrent, were explored. The numerical simulation and experimental results were in good agreement with those of the modified theoretical model. The measurement of in-plane vibrations of nanoscale amplitude in the frequency range of thousands of hertz was achieved.

## 2. Theoretical Analysis

**Figure 1:**
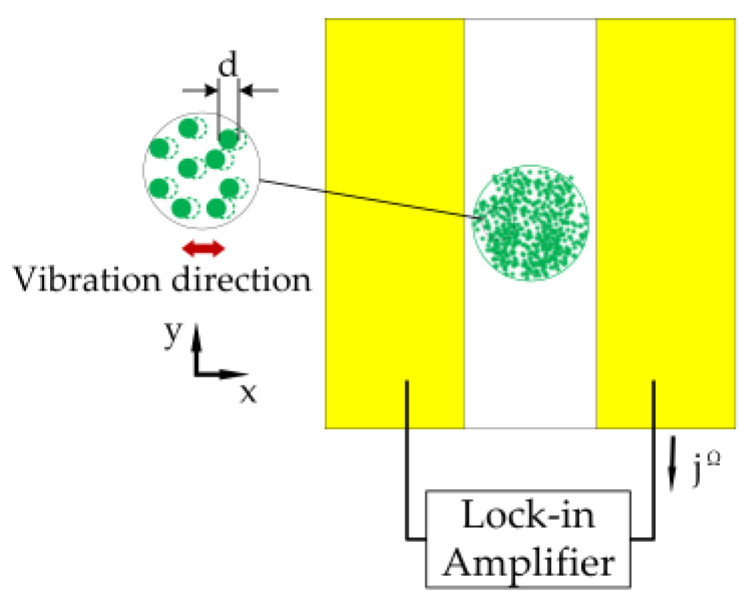
Principle of in-plane vibration measurements based on GaAs.

The photo-emf effect is a weak non-linear optical phenomenon. A photorefractive crystal is used to receive scattered light from a remotely measured object with vibration information (as shown in Figure 1). A short-circuited crystal is illuminated by a speckle pattern with a speckle size of *d*. When the oscillatory movement of a speckle with a certain frequency occurs, a photocurrent is generated. The photocurrent is caused by the generation of charge carrier distribution in the conduction or valence band and the formation of a space charge field in the photoionization process; the charge carrier distribution will vibrate with the optical pattern vibration. If the time required for the spatial charge field buildup of the material is significantly lower than the vibration frequency, the vibration of the photogenerated charge carrier density can be considered to occur in the static space charge field. This behavior causes a vibrational phase shift between the static space charge field and the carrier density, resulting in the generation of a non-steady-state photocurrent.

The intensity of a speckle pattern can be approximated by considering it as a set of Gaussian beams whose diameters are normally distributed around a diameter equal to the average speckle size. Each Gaussian beam represents a speckle particle, and the diameters of these Gaussian beams are approximately equal to the average speckle size [35]

(1) $$d=1.22\lambda \frac{d_{i}}{D_{p}},$$

where *d* is the subjective speckle size, *d_i_* is the image distance, *λ* is the wavelength, and *D_p_* is the imaging system aperture. The light intensity of speckled particles can be expressed as

(2) $$I=I_{0}e^{-\left(x^{2}+y^{2}\right)},$$

where w= *d/*2, $x=X/\sqrt{2}w$, and $y=Y/\sqrt{2}w$ are the coordinates normalized by the spatial coordinates *X* and *Y*, respectively; w= *d/*2 is the Gaussian beam radius. When the measured object vibrates along the transverse direction *x* with amplitude *δ* and angular frequency Ω, the speckle light pattern follows the measured object vibration with angular frequency Ω and normalized amplitude A=Mδ/w, where *M* is the magnification of the imaging system. Equation (2) changes to

(3) $$I=I_{0}e^{-[\left(x+A\sin\Omega t{)}^{2}+y^{2}\right]},$$

Assuming that the material response time *τ_sc_* is significantly longer than the period of the vibration pattern $2\pi /\Omega \;\left(\tau_{sc}\Omega \gg 1\right)$, which is significantly longer than the photoelectron lifetime τ (τΩ≪1), and considering cyclic frontier conditions, the average current density along the electrode space *L* can be obtained. The time-dependent term is as follows [30].

(4) $${\bar{j}}_{xt}\left(t\right)=\frac{j_{D}}{l}\int_{-\frac{l}{2}}^{\frac{l}{2}}e^{-[\left(x+A\sin\Omega t{)}^{2}+y^{2}\right]}\frac{E_{0}/E_{D}+\frac{\Omega }{2\pi }\int_{-\frac{2\pi }{\Omega }}^{\frac{2\pi }{\Omega }}e^{-[\left(x+A\sin\Omega t{)}^{2}+y^{2}\right]}\;\left(2x+2A\sin\Omega t\right)dt}{\frac{\Omega }{2\pi }\int_{-\frac{2\pi }{\Omega }}^{\frac{2\pi }{\Omega }}e^{-[\left(x+A\sin\Omega t{)}^{2}+y^{2}\right]}dt+R_{d}}dx,$$

where $j_{D}=e\mu E_{D}N_{0}$ and $E_{D}=D/\mu w$. *μ* and $D=k_{B}T\mu /e$ are the mobility and diffusion coefficients of the charge carriers, respectively; *k_B_* is the Boltzmann constant; *T* is the absolute temperature; $E_{0}=j_{0}/e\mu N_{0}$ is the external electric field applied between the two electrodes; *e* is the electronic charge; *j*_0_ is the average current density along the *x*-direction *x*; $R_{d}=N_{d}/N_{0}$ is the dark-to-bright conductivity parameter; and l=L/w.

**Figure 2:**
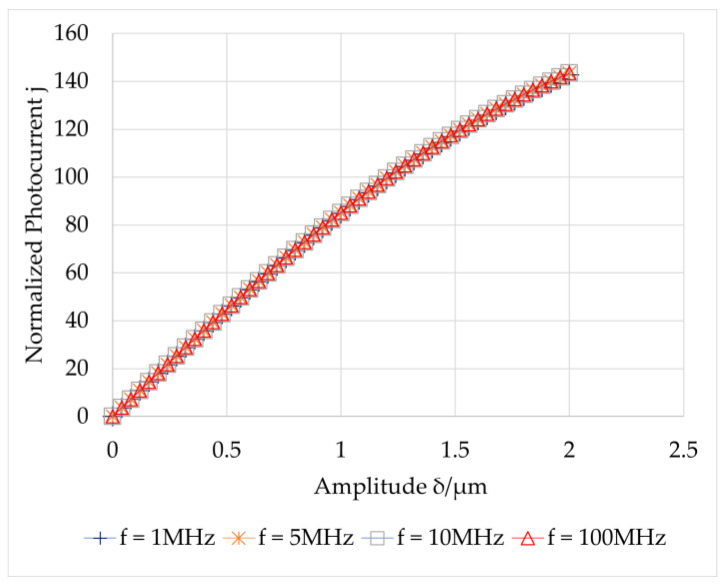
Relation between the photocurrent and vibration amplitude under *τ_sc_*Ω >> 1.

The photocurrent was obtained by the average current density multiplied by the area of the transverse electrode. Figure 2 shows the relationship between the amplitude and photocurrent at different high frequencies, which satisfy $\tau_{sc}\Omega \gg 1$.

If *τ_sc_* is not significantly longer than the vibration period 2π/Ω, Equation (4) should be modified to

(5) $${\bar{j}}_{xt}\left(t\right)=\frac{j_{D}}{l}\int_{-\frac{l}{2}}^{\frac{l}{2}}\begin{array}{c} \frac{E_{0}/E_{D}+\frac{\Omega }{2\pi }\int_{-\frac{2\pi }{\Omega }}^{\frac{2\pi }{\Omega }}e^{-[\left(x+A\sin\Omega t{)}^{2}+y^{2}\right]}\;\left(2x+2A\sin\Omega t\right)dt}{\frac{\Omega }{2\pi }\int_{-\frac{2\pi }{\Omega }}^{\frac{2\pi }{\Omega }}e^{-[\left(x+A\sin\Omega t{)}^{2}+y^{2}\right]}dt+R_{d}} \end{array}\;e^{-[\left(x+A\sin\Omega t{)}^{2}+y^{2}\right]}\frac{\tau_{sc}\Omega }{\sqrt{1+{\left(\tau_{sc}\Omega \right)}^{2}}}dx,$$

where $\tau_{sc}=\frac{\varepsilon \varepsilon_{0}}{\sigma_{0}}$, *ε* is the dielectric constant, *ε*_0_ is the vacuum permittivity, and *σ_0_* is the photoconductivity of the crystal [32].

The response time *τ_sc_* depends on the material itself, as well as on the incident light intensity and geometric parameters, such as the speckle size in this case. The formula of *τ_sc_* should be further clarified for sufficiently accurate photocurrent simulation. By analogy with the response time expression in the existing interferometric measurement mode [37], the specific formula for *τ_sc_* in the speckle measurement mode can be expressed as

(6) $$\tau_{sc}^{-1}=\frac{\sigma_{0}}{\varepsilon \varepsilon_{0}}=\frac{e\mu \alpha P_{l}}{\varepsilon \varepsilon_{0}h\nu {\left(\frac{L}{L_{D}}\right)}^{2}}=\frac{\alpha P_{l}}{\varepsilon \varepsilon_{0}\left(\frac{h\nu }{e}\right)\left(\frac{k_{B}T}{e}\right){\left(\frac{L}{L_{D}}\right)}^{2}},$$

where *α* is the optical absorption coefficient, *h* is Planck’s constant, *ν* is the light frequency, $P_{l}$ is the incident light power, and $L_{D}=$ *d/*2π is the average diffusion length of photoinduced carriers.

## 3. Numerical and Experimental Results 

### 3.1. Experimental Arrangement

**Figure 3:**
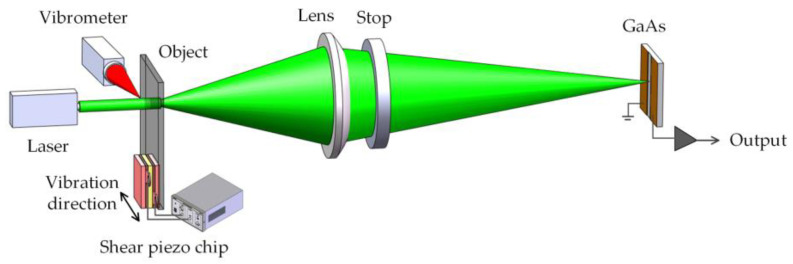
Schematic of experimental system.

The experimental setup is illustrated in Figure 3. A GaAs crystal (10 mm × 10 mm × 0.8 mm, crystal orientation [100], Molecular Technology GmbH, Berlin, Germany) with two parallel striped gold electrodes with an inter-electrode space of 2 mm on the front surface served as the detector. Its main properties include a dielectric constant of 13.1, mobility of 5.2 × 10^3^ cm^2^/(V·s), lattice constant of 0.56534 nm, and band gap of 1.4 eV. The measured object was a small and thin scattering glass plate, which was firmly adhered to a shear piezoelectric chip (PL5FBP3, Thorlabs, Newton, New Jersey, United States) and could generate transverse vibrations. 

The scattered glass plate was illuminated by a laser beam with a wavelength of 532 nm, 200 mW. The transmitted light was collected through a lens with a focal length of 50.8 mm, and the speckle pattern was obtained on the GaAs detector. A stop placed adjacent to the lens was used to control the transmitted light aperture. When the vibration frequency is sufficiently high, the space charge field will not match with the photoconductive phase of GaAs, resulting in a photocurrent of the corresponding frequency. The photocurrent generated by the GaAs was measured using a lock-in amplifier. A laser vibrometer (PDV100, Polytec, Baden-Württemberg, Germany) was placed perpendicular to the optical measuring path. The measuring light hits the side of the object to obtain the corresponding out-of-plane vibration, which is also in-plane vibration. This information was used to verify the in-plane vibration and calibrate the vibration information of the measured object.

**Figure 4:**
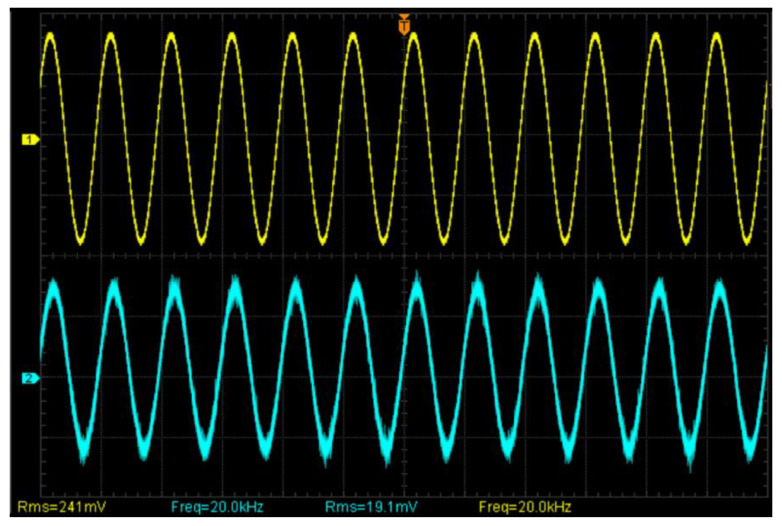
Time-domain comparison measurement results at 20 kHz.

The shear piezoelectric plate was driven by 200 V at a 20 kHz sinusoidal voltage. The time-domain comparison measurement results are shown in Figure 4. Signal 1 (yellow) was obtained using PDV100, and signal 2 was obtained using GaAs (blue). The measurement results are consistent, which verifies the feasibility of the system.

**Figure 5:**
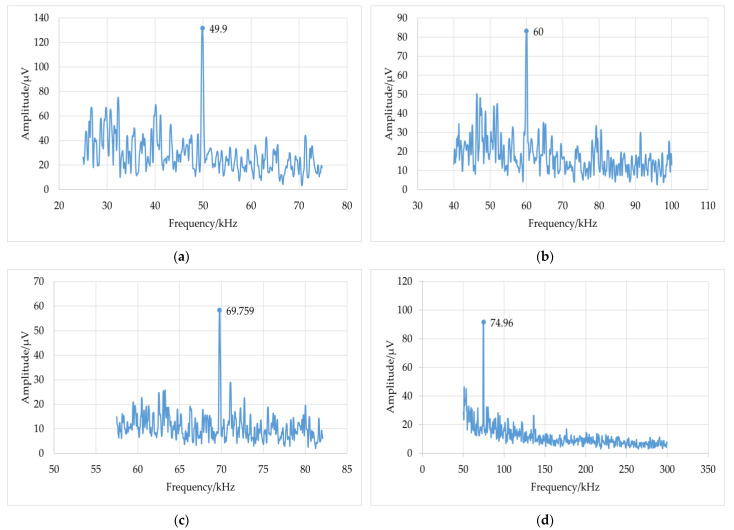
Measurement results of the system at different frequencies: (**a**) 50 kHz; (**b**) 60 kHz; (**c**) 70 kHz; (**d**) 75 kHz.

The small in-plane vibrations at 50 kHz, 60 kHz, 70 kHz, and 75 kHz were measured separately using this system, and the frequency-domain signals were obtained using a spectrometer. The results are shown in Figure 5. It can be observed that the measured vibration frequency is consistent with the signal frequency loaded on the shear piezoelectric chip. 

### 3.2. Results

The amplitude of the measured object was calibrated with the PDV100 laser vibrometer. The effects of the amplitude and frequency of the vibration, imaging magnification of the measuring system, and average speckle size of the measuring light on the induced photocurrent were explored.

#### 3.2.1. Effect of Vibration Amplitude

**Figure 6:**
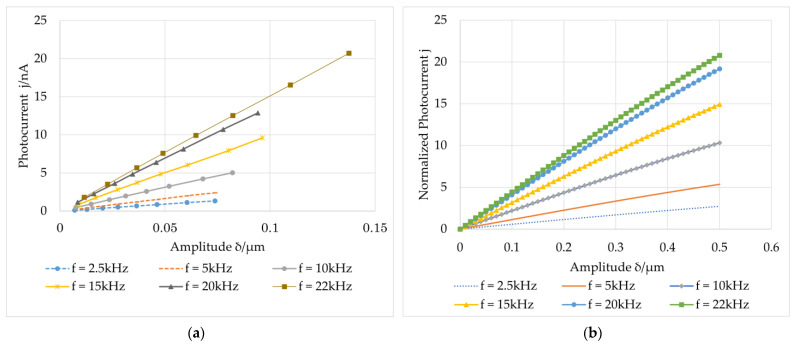
Relation between amplitude of the first harmonic of the photocurrent and amplitude of the vibration at different vibration frequencies: (**a**) experimental results; (**b**) theoretical simulation results.

The aperture diameter was set to *D_p_* = 50.8 mm, the lens focal length was *f* = 50.8 mm, and the object distance (from the diffusing element to the lens) was *d_s_* = 76.81 mm. We calculated the image distance (from the lens to the crystal) to be *d_i_* = 150 mm and the average speckle size to be *d* = 1.961 μm. At the same frequency, the amplitude of the measured object was changed by varying the input voltage of the shear piezoelectric chip. The measurement results and theoretical simulation results are shown in Figure 6.

As shown in Figure 6a, under the same vibration frequency, the induced photocurrent increases with increasing amplitude, which is the same as the simulation curve in Figure 6b. It can also be observed that the measurement system can measure in-plane vibrations of nanoscale amplitude. Although the response time is affected by the incident light intensity and the speckle size, GaAs is a fast response material. $\tau_{sc}\Omega \gg 1$ can be met when the vibration frequency reaches several megahertz or even tens of megahertz. According to the previous statement, the photocurrent is independent of the frequency and response time in this case. For a low vibration frequency of kHz, as shown in Figure 6, $\tau_{sc}\Omega \gg 1$ is not satisfied. When the vibration amplitude is small enough compared to the speckle size, the photocurrent is larger at higher frequencies and shows a monotonic increasing relationship.

#### 3.2.2. Effect of Vibration Frequency

**Figure 7:**
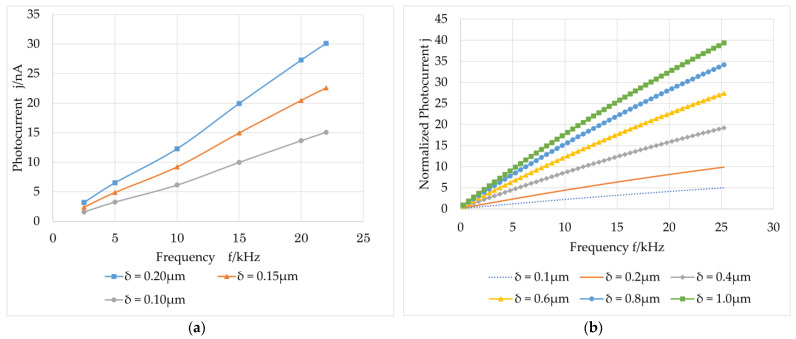
Relation between the amplitude of the first harmonic of the photocurrent and vibration frequency at different vibration amplitudes: (**a**) experimental results; (**b**) theoretical simulation results.

Under the same conditions that were applied in the previous experiment and the same voltage drive, different frequencies were loaded on the shear piezoelectric chip. Experiments and relevant theoretical simulations were performed to determine the relationship between the vibration frequency and photoinduced current, as shown in Figure 7. 

According to Figure 7a, the photocurrent increases with increasing vibration frequency, which is the same as the tendency of the simulation results in Figure 7b. When $\tau_{sc}\Omega \gg 1$ is not satisfied, the photocurrent will monotonously increase with the vibration frequency. 

#### 3.2.3. Effect of Imaging Magnification 

The magnification of the imaging system was $M=d_{i}/d_{s}=\frac{d_{s}}{d_{s}-f}=\frac{d_{s}-f}{f}$, and the average speckle size was $d=1.22\lambda \frac{d_{i}}{D_{p}}$. The image distance *d*_i_ must be controlled to maintain the average speckle size *d* = 1.961 μm while changing the imaging magnification. 

**Figure 8:**
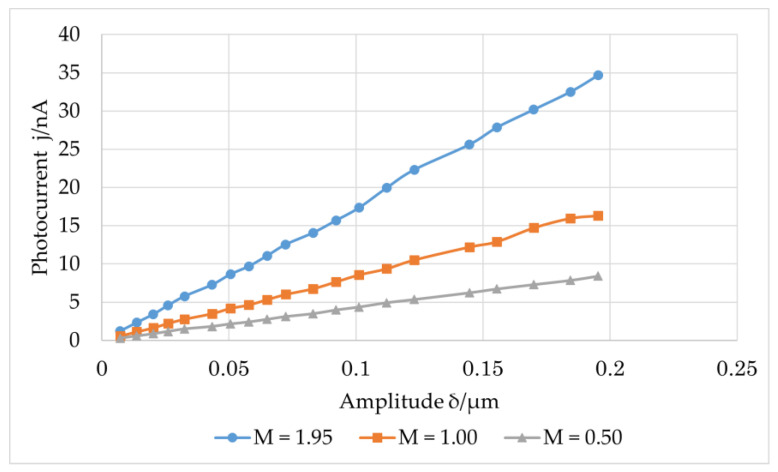
Relation between the amplitudes of the first harmonic of the photocurrent and vibration at different magnifications.

The vibration frequency was maintained at 22 kHz, and the focal length of the lens was varied to obtain different imaging magnifications. The vibration amplitude of the speckle changed with the imaging magnification. The relationship between the photocurrent and in-plane vibration amplitude of the shear piezoelectric chip is shown in Figure 8, where the magnification M of the imaging system is 0.5, 1, and 1.952. 

As shown in Figure 8, when the imaging magnification of the measurement system increases, the photocurrent also increases. This behavior occurs because the measured object will be magnified on the image plane after passing through the imaging system, and the amplitude of its vibration will also be magnified; thus, the photocurrent generated will also increase.

#### 3.2.4. Effect of Average Speckle Size

**Figure 9:**
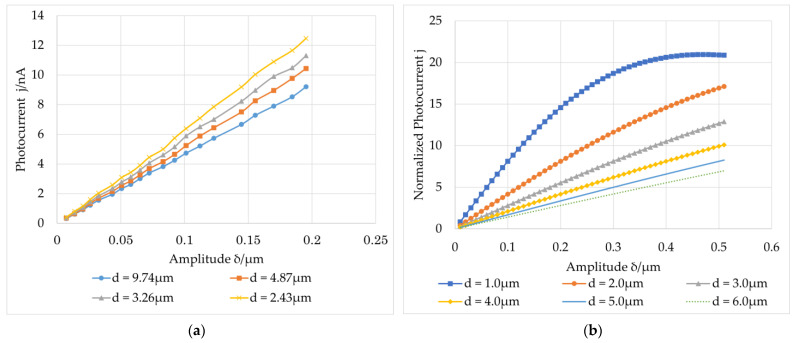
Relation curve between the amplitude of the first harmonic of the photocurrent and amplitude of the vibration at different average speckle sizes: (**a**) experimental results; (**b**) theoretical simulation results.

The average speckle size was changed by changing the aperture of the imaging system. The focal length of the lens was 50.8 mm. A variable stop was placed behind the lens to change the size of the transmitted light. An optical attenuator was used to maintain the average intensity of the image surface unchanged. The experimental results obtained by adjusting the aperture to 10, 20, 30, and 40 mm are shown in Figure 9a, and the theoretical simulation results are shown in Figure 9b.

It can be observed that the smaller the average speckle size, the larger the output photocurrent. This finding is consistent with the simulation results shown in Figure 9b. As previously mentioned, the maximum value of *j^ω^* will appear when the amplitude *δ* approaches the speckle radius w if $\tau_{sc}\Omega \gg 1$. However, the material response time should be considered for a case that does not meet $\tau_{sc}\Omega \gg 1$. The relationship between the photocurrent and amplitude in this case can be obtained by approximately estimating the response time. The photocurrent also monotonously increased with the in-plane vibration amplitude.

## 4. Conclusions

In this study, we developed a speckle measurement method for in-plane vibrations based on the photo-emf effect of crystal GaAs. The relevant theoretical models were utilized. Through experimental and theoretical simulation, the measurement sensitivity of nanoscale vibration was confirmed and the influences of the vibration amplitude, the vibration frequency, the imaging magnification, and the average speckle size on the first harmonic of the induced photocurrent when $\tau_{sc}\Omega \gg 1$ was not satisfied were determined. The accuracy of the supplementary response-time calculation formula was verified. This study provides a theoretical simulation and experimental basis for the application of GaAs in the measurement of in-plane, high-frequency submicron vibrations.

## Data Availability

Not applicable.
